# Reflections on Neurofeminism and Intersectionality Using Insights From Psychology

**DOI:** 10.3389/fnhum.2021.684412

**Published:** 2021-09-29

**Authors:** Annie Duchesne, Anelis Kaiser Trujillo

**Affiliations:** ^1^Department of Psychology, University of Northern British Columbia, Prince George, BC, Canada; ^2^Gender Studies in STEM, Institute of Computer Science, University of Freiburg, Freiburg, Germany

**Keywords:** sex/gender, neuroscience, feminism, intersectionality, psychology, epistemology, social structures, social justice

## Abstract

Intersectionality contends that sex/gender is constituted of and with other social categories, and that the social structures giving rise to inequality should be addressed in research. This is a powerful and important perspective from which to investigate the processes and consequences of social group memberships, one which has been overlooked by most neuroscientific research. In particular, neurofeminism, a field of critical neuroscience that challenges neuroscientific assumptions, methods and interpretations of data that reinforce sexism, has ignored intersectionality to date. In contrast, research in the field of psychology has been engaging with intersectionality for more than a decade. In reflecting on how intersectionality has advanced feminist research in psychology, this paper provides a critical analysis of potential novel research avenues for neurofeminism. We identify three main research themes guided by intersectionality. The first theme involves research centered on understanding the *socio-structural causes* of health inequalities experienced by individuals with intersecting marginalized social identities; the second concerns research addressing the *psychological processing of social group memberships* that underlies the enactment of systemic discriminatory practices; and the third theme comprises intersectionality research that aims to challenge *psychological epistemology*. Drawing parallels between the fields of psychology and neuroscience, we explore the potential benefits and risks of advancing an intersectionality-informed neurofeminism.

## Introduction

Neurofeminism is the feminist practice and criticism of neuroscience. Neurofeminists challenge research practices, including assumptions, methods, and interpretations of data that reinforce sexism by treating neuroscientific knowledge as acultural, apolitical, and sexually dichotomic (Kuria and Hess, 2011; Bluhm et al., 2012; Schmitz and Hoppner, 2014). To overcome the flaws of traditional sex/gender^1^ neuroscience, neurofeminist work has developed alternative conceptual (e.g., the mosaic brain, Joel, 2011; Joel et al., 2015) and methodological (e.g., brain size correction, Rippon et al., 2014; Sanchis-Segura et al., 2020) neuroscientific approaches to studying sex/gender. These contributions highlight the context of neuroscience as a discipline (Fine, 2010; Roy, 2012; Jordan-Young and Karkazis, 2019), recognize the constraining role of sexed/gendered experiences in shaping sex/gender development (Fausto-Sterling, 2021), address the role of sex/gender in brain structure and function (Eliot, 2011), and understand sex and gender as fundamentally intertwined (Kaiser, 2012). One important contribution of neurofeminism to date has been to expose methodological and conceptual biases within neuroscientific research postulating that sex/gender differences in behavior are fundamental and caused by “hard-wired” dissimilarities between women’s and men’s brains (Fine, 2012; Joel and Vikhanski, 2019; Jordan-Young and Karkazis, 2019; Rippon, 2019; Eliot et al., 2021). Another important contribution has been the demonstration that certain sexed/gendered *behaviors*, irrespective of assigned sex/gender at birth, can induce hormonal change (e.g., aggressive behavior increases testosterone, nurturing behavior decreases testosterone; van Anders and Gray, 2015) reinforcing the notion that sex-based biological differences, if any, are influenced by socio-cultural differences such as behavioral expression. Finally, neurofeminists have provided numerous recommendations related to the epistemological assumptions, language use, postcolonial constraints, and the categories and research methods employed to conduct sex/gender-related neuroscientific investigations (Einstein, 2012; Kuria, 2014; Rippon et al., 2014; Roy, 2018; Duchesne et al., 2020).

Despite these successes, the feminist approach to sex/gender-related neuroscientific research remains in the margins of the field, particularly since national funding agencies have incentivized sex-segregated biological research (as discussed in Eliot and Richardson, 2016; Joel and McCarthy, 2017; Gungor et al., 2019). For instance, in 2016, the National Institute of Health started requiring awardees to account for sex as a biological variable (SABV) in all stages of their research (design, analysis, and reporting) in vertebrate animals and humans (National Institutes of Health (NIH)., 2015). Currently, calls for SABV-based neuroscience abound (e.g., Bale and Epperson, 2017; Bath, 2020; Bhargava et al., 2021; Shansky and Murphy, 2021). In recent years, an increasing number of feminist scholars have advocated for bioscience researchers to engage with intersectionality as a theoretical framework^2^ that could aid in generating socially contextualized and reflective biological knowledge, and provide a counternarrative to other essentializing and risk-oriented explanations in biomedicine (for a review, Hankivsky et al., 2017; DeBlaere et al., 2018; Shattuck-Heidorn and Richardson, 2019; Jacke and Palm, 2020). However, to date, intersectionality remains largely overlooked in the design, analysis, and interpretation of sex/gender-related neuroscientific research. As this special topic aims to advance the development of critical investigative approaches in sex/gender and the brain that are grounded in plurality, we explore whether and how intersectionality can provide novel research avenues for neuroscience, and in particular, for neurofeminism.

Rooted in Black feminist activism, intersectionality as a theoretical framework states that sex/gender is constituted of and with other discriminatory social categories (Shields, 2008; DeBlaere et al., 2018; Mays and Ghavami, 2018). First articulated in qualitative legal research to deconstruct the sexed/gendered experiences of African American women (Crenshaw, 1989, 1990), intersectionality as a theoretical framework currently informs research across several disciplines that investigate various processes involved in experiences of social injustice emerging from intersecting group memberships (e.g., De Vita et al., 2016). For more than a decade, sex/gender research in psychology has been informed by intersectionality (Shields, 2008). As this literature grows there has been much debate as to which conceptual (McCormick-Huhn et al., 2019), methodological (Else-Quest et al., 2006; Bowleg and Bauer, 2016; Scott and Siltanen, 2017), and epistemological (Warner et al., 2016) approaches to conducting psychological research best align with an intersectionality framework.

The current field of sex/gender-related neuroscientific study is largely uninformed by an intersectionality perspective, treating sex/gender as a category orthogonal to other social group memberships. Adopting an intersectional approach means adopting a commitment to understanding the interdependence of social group memberships beyond conventional factorial interactive analyzes of interdependence of social group memberships. In this paper, we draw from a breadth of psychological research to explore potential benefits and risks of using intersectionality in neuroscience. Specifically, we identify three psychological research themes that differ both in their use of intersectionality, and in the domain of psychology under investigation. The first approach to employing intersectionality in psychological research focuses on understanding *the socio-structural causes of health inequalities* in individuals with intersecting marginalized social identities. The second approach uses intersectionality to interrogate the *psychological processing of social group memberships* that underlies the enactment of systemic discriminatory practices. Finally, the third approach employs intersectionality to interrogate how psychological knowledge is produced and understood, and in doing so, *challenges psychological epistemology*. Each research theme will be compared with neuroscientific research informed by intersectionality, if any.

## Research Theme #1: How Social Structures Create Health Inequality in Individuals With Intersecting Social Identities

One theme of psychological research informed by intersectionality focuses on delineating the social structures responsible for health inequities experienced by individuals with marginalized intersecting social identities. Social structures are defined as the social layouts of a society that arise from and subsequently constrain people’s actions, resulting in the categorization of individuals in groups through normative sets of roles, functions, meaning, purpose, and power dynamics (Haslanger, 2016). Socio-structural factors are a source of influence at all levels of society, including laws, policies and practices, economic characteristics, occupations, and familial organization. Psychological research conducted with the goals of (1) exposing the complexity of oppressive social structures related to group membership and (2) understanding the health ramifications of such structures, uses varied quantitative and qualitative methodologies. Such research is centered on populations that are often hidden from major analysis and health inequality frameworks, and avoids notions of simplistic, additive social categorization (e.g., categorizing people by race, class, or sex/gender) by conceptualizing social group membership categories as *interdependent* rather than independent (Bowleg, 2008; Warner, 2008). Importantly, the interpretation of research findings within this approach is oriented toward concrete action for social change and justice. In sum, this type of psychological research employs intersectionality to guide the development of the research problem, the selection of methods, the study population and the data interpretation, in order to produce psychological knowledge about health inequalities that is contextualized within an understanding of oppressive socio-structural power dynamics, with the goal of dismantling them (Bowleg, 2008).

A recent publication by Kteily-Hawa et al. (2019), highlights the importance of this type of research for elucidating complex interactions between social categories in relation to health outcomes (Kteily-Hawa et al., 2019). This study investigated how oppressive social structures associated with immigration experiences increase health vulnerability in South-Asian women living with HIV in Canada. The authors conducted interviews and thematic analyzes focused on how power relations, emotional relations, social norms and sexed/gendered divisions of labor intersect to create a unique context that increases the risk of HIV (Kteily-Hawa et al., 2019). Their findings revealed that sexed/gendered roles within the household reinforced male control over the division of labor at home, and that these dynamics were in turn reinforced by immigration experience. Similarly, English et al. (2020) investigated socio-structural factors related to psychological health and health behavior outcomes within HIV-positive, Black sexual minority men (SMM), and demonstrated how history of incarceration, recent police arrest, and experiences of discrimination by police and other law enforcement interact to predict sexual behaviors related to HIV risk, psychological distress, and the motivation to seek prophylactic treatment (English et al., 2020). As highlighted by the authors, the unique carceral and law enforcement experiences and health correlates of Black SMM, a population at increased risk of incarceration in the United States, are often overlooked when their data are aggregated with those of Black heterosexual men or White SMM, rendering Black SMM an intersectionally invisible population. This study revealed negative health consequences of experiences with law enforcement for the Black SMM community in demonstrating that prior incarceration history, police and law enforcement discrimination, and recent arrest all showed direct and indirect relationships to worse psychological health outcomes. By exposing the socio-structural factors associated with health inequality within certain group memberships, this type of intersectionality research provides an understanding of health that is directly linked to power dynamics, and offers an approach to studying health and wellness that has the capacity to promote social change.

Importantly, this type of intersectionality research differs meaningfully from research that focuses on health outcomes *as a function of* broad, decontextualized social categories. Labeled “flattened” intersectionality, this latter type of research tends to explore the interaction between broad social categories (e.g., sex/gender, race, and class) without any assessment of socio-structural or other contextual factors (e.g., discrimination), or in other words, treats social categories as fixed determinants outside of their socio-historical oppressive context (Warner et al., 2016), and thus avoids dealing with the “latent” issue of inherent socio-structural power relations. In using a “flattened” approach to intersectionality, the focus of the explanation becomes the individual. This shift in focus occurs at the expense of interrogating and ultimately dismantling the socio-structural power imbalances that underlie health inequality. By decontextualizing social categories from their socio-political structures, flattened intersectionality research leaves room for essentialist explanations (e.g., social selection explanation, Mackenbach, 2005), and with that, the possibility of reinforcing oppressive structures through ignoring, and thereby masking, their contribution to a psychological or other health-related phenomenon (examples reviewed in Buchanan and Wiklund, 2021). As recently described by Buchanan and Wiklund (2021), flattened intersectionality comprises a large portion of contemporary intersectionality research in psychology, which the authors attribute to exclusionary epistemic practices by “mainstream psychology” (epistemology is further discussed in section III; Buchanan and Wiklund, 2021). In contrast, intersectionality research that works to understand the complex socio-structural liberative and oppressive contexts of social group memberships moves away from broad categories and individual-centered explanations by explicitly positioning the roots of health inequality within social systems.

Neuroscientific research that studies the neural ramifications of health inequalities tends to focus analysis on a single group membership. For instance, the neural correlates of social class, or more specifically of poverty, are commonly investigated in neuroscience. Such studies have documented numerous associations between socioeconomic status (SES) and the function and structure of the developing brain (Hackman and Farah, 2009; McDermott et al., 2019). However, while this research characterizes brain correlates of oppressive economic conditions, it does not consider the social experiences and consequences of poverty as interdependently related to other social group memberships, and tends to “detach” material poverty from its oppressive socio-political context. Like the flattened intersectionality research described above, this kind of neuroscientific research inadvertently promotes essentialist and deterministic interpretations of brain data. This apparent paradox has been explored in a recent publication by Pitts-Taylor (2019): “most of the studies I reviewed propose that the effects of social inequality can become entrenched in the brain, shaping future neurobiological, cognitive, and even socioeconomic trajectories. In other words, they reify and ‘fix’ the phenotype” (Pitts-Taylor, 2019). Without accounting for socio-structural factors, researchers risk reinforcing the view that poverty persists due to cognitive “inferiority” rather than as a complex outcome arising from numerous avenues of social inequality.

To date, we are aware of one neuroscientific study examining the role of socio-structural context within a population characterized by multiple marginalized group memberships. Thames et al. (2018) demonstrate that the reported experiences of social adversities (racial/ethnic discrimination and childhood SES) corresponded with both structural brain differences and worse learning and memory performance (Thames et al., 2018). While this study broadly focused on different types of social adversity, its findings also captured how, in HIV-positive populations, the intersection of race- and class-based structural oppression is associated with neural and cognitive impairments. In their critical analysis of neuroscience, neurofeminists have emphasized that critical race analysis must be considered in any investigation aiming to understand and ultimately dismantle inequitable sexed/gendered conditions (Roy, 2012; Kuria, 2014; Rippon et al., 2014), and as our discussion highlights, research in neuroscience that is informed by intersectionality must expand its focus beyond sex/gender and race to include a wider spectrum of intersecting and marginalized identities. With the exception of a recent pain study conducted with Somali-Canadian women with female genital cutting (further detailed below, Perovic et al., 2021), to date, there are no neurofeminist parallels to this type of research (Fitsch et al., 2020).

In light of these observations, how can intersectionality advance neurofeminist work? First, explicitly approaching sex/gender as interdependently constituted of and with other social group memberships is a critical area for advancement. Second, increased focus should be placed on conducting research with populations of women and sex/gender diverse people that, because of their marginalized group memberships, are often rendered invisible. However, as mentioned, research that addresses intersectionality only at the level of individual identity is severely lacking and risks reinforcing oppressive social structures through ignoring the impact of these structures on health. It is critical for neurofeminists to formulate *how* specific socio-structural power dynamics may contribute to or fully explain previously observed sex/gender-related brain health inequalities. Only after identifying these socio-structural dynamics can researchers begin transforming these socio-structural factors, policies and practices, occupations, laws, familial organization, racial minority status, and economic characteristics into operationalized measures that can be incorporated into neuroscientific research designs. This is a foundational step toward advancing neurofeminism, and requires going beyond what has been articulated in neuroscience to date. Finally, researchers must consider selecting methodologies and analytical approaches that allow for the socio-historical contextualization of oppression and privilege (discussed further in section III). Sex/gender neuroscience research guided by intersectionality as articulated in this section will further contribute to understanding health outcomes contextually rather than centering on individual, deterministic risk factors.

In this section, we considered the importance of intersectionality as a framework for understanding outcomes related to health inequality as complex, contextualized phenomena arising in part from oppressive socio-structural power imbalance rather than individual risk alone. In the following section, we explore research that interrogates the psychological processes by which socio-structural oppressive attitudes and behaviors may arise.

## Research Theme #2: How Individuals Process Intersecting Social Identities

How do people process and understand information related to intersecting social categories? A second theme of psychological research informed by intersectionality relies on quantitative methodologies to provide an understanding of how information-processing related to different social categories may underlie processes of social discrimination. Primarily, this research theme focuses on representation of intersected social identities at the level of the stimulus bank of a study, and less on representation within a participant sample. In other words, this type of psychological research employs intersectionality to guide the development of the research question, while relying on traditional psychological approaches to study design, analysis, and interpretation of data.

Prior to the integration of intersectionality in psychology, psychological research examined racialized or sexed/gendered variables as independent stimulus categories. Using this type of categorization, abundant work exists on what was first called “race recognition” and later “racial bias” research, in which the aim was to measure the relative contributions of automatic (i.e., unconscious or unintentional) and controlled (i.e., conscious or deliberate) processing to a racialized phenomenon of study (Dasgupta and Greenwald, 2001). The assumption of much of this research (and indeed, of psychological science broadly) is to understand “fundamental” processes, and as such, the universality of these processes across individuals is often implicitly assumed. Such hidden assumptions of universality are reflected in the overwhelmingly common use of homogeneously Western Educated Industrialized Rich Democrats participant samples. Despite its possible contributions to our understanding of the psychological processes underlying discrimination, this approach of investigating how psychological processes and experience can be understood “in general” without regard to socio-structural context leads to research findings that partially bind results to a normative population and support the unequal power dynamics of existing societal structures through the uncritical reproduction of the dominant normative perspective.

**FIGURE 1:**
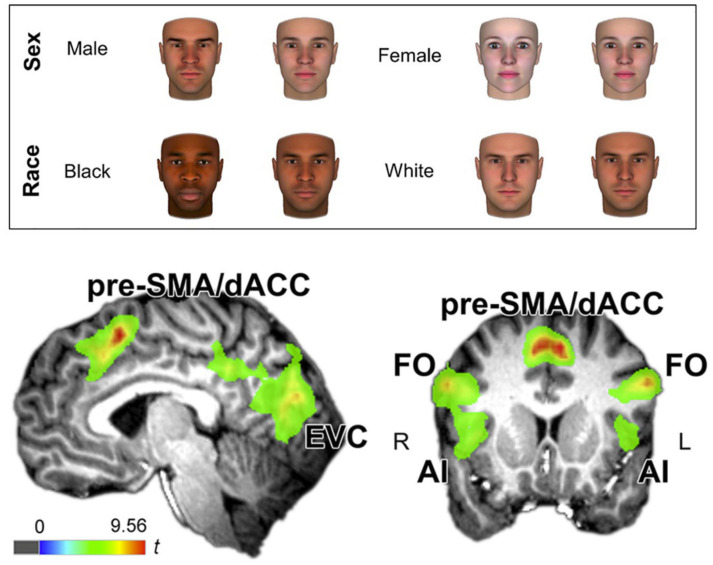
From Stolier and Freeman (2017) (CC-BY). Above: This example shows the use of stimuli in Cognitive Neuroscience. It demonstrates the challenge when attempting to visualize categories of social identity and it also demonstrates how these attempts reify structural power because, as shown, here, the category of “Black woman” misses it again to be shown. Below: The brain images show the neurobiological correlates of “atypicality”. Participants were presented with faces that had to be categorized corresponding to their “gender and racial typicality”. Activation was found in the cingulo-opercular network consisting of presupplementary motor area and dorsal anterior cingulate cortex (pre-SMA/dACC) and, centrally, the anterior insula/ and the frontal operculum (AI/FO). This pattern of activation suggests the engagement of conflict monitoring when atypical faces are shown. R: right, L: left. EVC: early visual cortex, specific brain activation discussed elsewhere in the cited paper showing the processing of target categories presented in the study.

Early research within this theme investigated the interacting effects of processing sex/gender and racial information using pictures of faces, again often through factorial designs in which categories are treated independently. In a seminal study by Goff et al. (2008), participants were presented with Black and White female and male faces. Results revealed a sex/gender categorization bias for stimuli depicting Black persons such that the perceivers judged both Black men and Black women as more masculine than White counterparts. Further, faces depicting Black women were rated as less attractive than White women, an effect that was mediated by ratings of masculinity. Numerous studies in face-based judgments have since expanded these findings by varying the racialization of the stimuli (Johnson et al., 2012; Hopper et al., 2014), the study’s target population, and age of participants being studied (Kim et al., 2015; Li and Tse, 2016; Lei et al., 2020). Importantly, the results of Goff et al. clearly expose a research bias toward white women in sex/gender-related social cognition research, based on a white majority (82%) of participants, as well as the erasure of Black women exemplified in Stolier et al.’s face stimuli visualization (2017), see Figure 1. This bias in the conduct and presentation of the research itself highlights a pressing need for psychology to begin operating within a theoretical framework that conceptualizes the perception of sex/gender as a process encompassing plurality contingent upon other social realities (Goff and Kahn, 2013).

Currently, this type of psychological research is seeing a development of novel methods that aim to integrate how social group memberships are processed and experienced. For instance, novel multiracial faces databases are being created, reflecting both the impact of intersectionality in the psychology and cognitive human neuroscience of face processing (Chaney et al., 2020; Chen et al., 2021) and the consequences of diversification in psychological samples. That said, the mere diversification of stimulus banks and participant samples does not address socio-structural power dynamics; what is considered “masculine” and what is considered “attractive,” are strongly informed by the socio-structural power dynamics that are commonly overlooked in these kinds of studies, which results in research that merely summarizes descriptively the very processes of discrimination for which it attempts to elucidate psychological mechanisms.

Alternatively, an increasing number of experimental studies use intersectionality to investigate the psychological processes at play in the experience (rather than perception) of intersecting social identities. For instance, in a study investigating how participants’ own sex/gender and race relate to perceived safety and threat cues in Black, Latina and white women, Chaney et al. (2020) demonstrated the transferability of threat, but also safety cues, from the racial to the sex/gender category – meaning, for instance, that Black and Latina women anticipated both racial and sex/gender discrimination from an identity threat stimulus that was designed to target only one of their stigmatized identity categories (Chaney et al., 2020). Similarly, when presented with an identity safety cue, the safety experienced in relation to one category is transferred onto the other category. By demonstrating at the psychological level how intersecting marginalized social identities confer disadvantage and advantage (e.g., experience of threat or safety) depending on the social situation, this research exposes the ramifications of power imbalance in social inequality. Taken together, these findings provide evidence and novel tools for an increased representation of the diversity of social group membership (e.g., databases), and even form the basis for potential, direct improvement of social interventions and advocacy policies.

Concerning neuroscientific approaches, abundant research exists within the field of facial processing and decision-making, but this research has not been informed by intersectionality. Extant studies have investigated the neural correlates of “social categories” (Wiese et al., 2008; George, 2016; Stolier and Freeman, 2017; Delplanque et al., 2019; Brooks et al., 2020), using narrow examinations of single constructs such as sex/gender, racial categorization, race-related prejudice or sex/gender stereotyping (e.g., Kaul et al., 2011; Senholzi et al., 2015; Mattan et al., 2018; Fisher et al., 2020). To our knowledge, only one study has investigated the neural correlates of face processing of multiple social group memberships in face processing.

In their paper “Neural pattern similarity reveals the inherent intersection of social categories,” Stolier and Freeman (2016, p. 795) suggest that the social categories of sex/gender, race, and emotion expression are “inherently intertwined” in the neural process of facial recognition. Their behavioral and fMRI experiments employing representational similarity analysis demonstrate that both the subjective perception and neural representation of social categories is contingent on participants’ social-conceptual knowledge of identity-related stereotypes. For instance, in emotional categorization, Black faces were disproportionately categorized as angry, while female faces were disproportionately categorized as happy. The subjective interdependency of social categories in face processing was also represented in differential brain activity within the orbitofrontal cortex and right fusiform cortex. Interestingly, visual similarities of image silhouettes or pixel-intensity did not fully explain the intertwined aspects of the social categories at the neural level, reinforcing the interpretation that it is the subjective, social-conceptual knowledge that underlies the brain’s processing of these identity categories. Findings from this study show that people’s social stereotypes about particular intersected identity categories are reflected in both subjective judgments and neural representation in a clearly interdependent manner, revealing a possible effect of social inequality in the neurobiology of face perception.

This study by Stolier and Freeman (2016), demonstrates both the strengths and weaknesses of this type of “intersectional” research. One strength is that, despite the study’s focus on the brain “basis” of intersecting social categories, the authors’ conclusion that subjective social stereotypes shape the neural processing of faces elevates a social interpretation of face processing over a purely biological interpretation, and thereby avoids the pitfalls of resorting to biological essentialism. The authors also recognized as a main limitation that their findings “are mute with respect to the origins of the stereotypical associations studied here” and suggest that these could result from cultural transmission and implicit learning (Stolier and Freeman, 2016, p. 797). They do not interrogate this finding any further; in this regard, they sidestep the question of whether these subjective stereotypes are “fixed” at the level of the brain or whether they can be changed, and instead suggest that future studies should aim to manipulate participant’s social stereotype in order to improve causal inference. Further, they do not discuss how socio-structural power dynamics may influence the development of stereotypical social categorization, thereby treating each of the categories as “neutral.” As a result, even when adopting an explicit focus on the contributions of subjective social-conceptual knowledge to processing of social categories, research that aims to localize distinct patterns of neural activity related to intersectional categories in the brain runs the risk of inadvertently biologically essentializing these categories, simply in a more multifaceted, “intersectional” manner than arises from the “traditionally” separated social categories. This significant stumbling block may be one reason that neurofeminists have skirted the issue of intersectionality to date (Fitsch et al., 2020). Despite these limitations, Stolier and Freeman’s work is nonetheless a contribution to the neurofeminist field as it provides support for the constitutive role of social experiences, in particular intersecting social group membership, in the subjective perception and neural processing of faces, and highlights that processing of intersectionality is not purely stimulus-driven.

Will engaging in this type of research be a fruitful avenue for neurofeminism? To counteract the limitations of this type of research, consideration for the interdependence of intersected identity categories needs to be contextualized within an understanding of socio-structural power dynamics. This includes an understanding of the relation between social group memberships and corresponding power differentials between researchers and participants. The social categories of sex/gender, race, and emotion are not neutral, independent categories within or across social group membership. Adopting an approach like that of Chaney et al. (2020), where the processing of social group membership is considered together with who is processing these social cues, will further expand our understanding of the context-contingency of processing group memberships. Finally, developing studies that not only manipulate social group stereotypes, as suggested by Stolier and Freeman, but also manipulate the social power dynamics, could provide new insight into the brain processing of sex/gender.

In this section, we highlight how the use of intersectionality in research that aims to understand the psychological and neurocognitive processing of social group memberships could lead to new research avenues in the neuroscience of sex/gender. However, the ways in which intersectionality is incorporated into this research is not without an important consideration of shortcomings. Given the difficulties that arise when trying to reconcile an inherently reductive, quantitative approach to producing generalizable knowledge about the brain (i.e., the approach that forms the foundation of the scientific method), it is unclear if critical neurofeminism can engage with this type of research without risking harm related to biological essentialization of “intersected” categories. In the following section we consider whether psychology or neuroscience can accommodate an intersectionality perspective at the epistemological level without inadvertently expanding notions of biological essentialism through harmful dimension reduction of social categories in the brain.

## Research Theme #3: How Epistemology Can Benefit From Intersectionality

A third type of research uses intersectionality to interrogate epistemologies in psychology. Instead of informing the selection of the research population (theme 1) or informing both the research questions and methodical considerations such as choice of stimuli (themes 1 and 2) and interpretation of findings (theme 1), here intersectionality is used to critically interrogate the foundations of knowledge production in psychology. By considering knowledge as political, embedded in power dynamics, and bound to human experiences, the intersectionality perspective on knowledge production is viewed as a critical process of continuous transformation (Marecek, 2016; Else-Quest and Hyde, 2016a, b; Grzanka, 2018; Collins, 2019; Rice et al., 2019). In line with previous work by feminist science and technology scholars and philosophers (Haraway, 1984; Longino, 1987; Fausto-Sterling, 2000; Schiebinger, 2001; Harding, 2006; Hammonds and Herzig, 2008; Subramaniam, 2009). This position on defining “knowledge” renders the knower’s social position a constitutive part of knowing, where knowing is an ever-changing process (Anderson, 2020). Because one’s social position constitutes a central element of what knowledge is, this position also informs how empirical inquiry can be or should be conducted within a particular knowledge domain. This idea stands in stark contrast to the positivist epistemologies that dominate much of psychological science, wherein observable evidence is the only form of defensible scientific findings, and only “facts” derived from the scientific method can support legitimate knowledge claims. Intersectionality research of this third type disrupts this assumption and related practices, and in doing so generates novel avenues for psychology (Warner et al., 2016).

In a recent publication, Settles et al. (2020) highlight epistemological points of rupture between an intersectional and psychological perspective on knowledge production. Conceptually, these ruptures are reflected in how intersectionality considers “generalizable” explanations of psychological knowledge to be probable *distortions* of the investigated phenomenon. Methodologically, intersectionality challenges the notion of psychological norms and their associated measurements in favor of modes of inquiry oriented toward diverse participants’ lived and historical experiences, especially when engaging in quantitative research (Bowleg and Bauer, 2016). Further, conceptual and methodological shifts are currently being observed in the involvement of the participant as co-creator of the research. Overstreet et al. (2020) suggest that research informed by intersectionality demands participant involvement in the development of the research question and methods, while also requiring the researcher to reflect on how systems of power may bias the assumptions and practice of psychological research. An intersectional perspective necessitates that psychological knowledge, theory, and research must be oriented toward social justice actions and goals, making social activism a central consequence of advancing psychological knowledge (Settles et al., 2020). To do so requires an interdisciplinary approach in order to adequately socio-historically situate the participants, the phenomenon, and the knowers. This approach to producing knowledge goes against the traditional structure of academia and psychology (Warner et al., 2016) and questions numerous foundational research practices in psychology.

Despite its rich conceptual and methodological ramifications, work that uses intersectionality to critically analyze psychological knowledge production tends to be devalued and is predominately absent from mainstream psychological literature. Settles et al. report that critical intersectionality research in psychology is subject to epistemic exclusion, wherein the research itself is marginalized and undervalued as contributing minimally to the advancement of psychological knowledge. This exclusionary practice translates to a general lack of interest, or else a perception that this work is inaccessible, which results in various bias-inducing practices such as the marginalization of intersectional work within specialized journals (Settles et al., 2020). This publication bias in turn leads to an epistemic bias in mainstream psychology, which results in the disproportionate propagation of less critically conducted, flattened intersectionality research of the sort commonly observed in the field of psychology (Bilge, 2013; Warner et al., 2016). Crucially, this form of epistemological exclusion also leads to even further biases in the broader culture of academia regarding both the value of this critical work and the recognition of those conducting it—scholars who often themselves occupy marginalized positions. Settles et al. (2020) state: “Our position as marginalized scholars due to our identities (gender, race, and sexual orientation) is what brings us to the work that we do, including the populations we study, the questions we ask, and the theoretical lens we use.” The challenges we face in the academy provide us with an insider perspective on the epistemic exclusion of intersectionality in psychology and the implications such exclusion has on academic careers, including our own. In relation to this exclusion, Cole also raises concerns that the burgeoning use of intersectionality in research contexts is increasingly disconnected from the lives and concerns of women of color, as are the contributions of Black women scholars (Cole, 2020). Committing to critical intersectionality research in psychology means risking that both your work and status as a scholar will be subject to exclusion and erasure, a position disproportionately experienced by minority scholars, who often face pressures to “mainstreamify” their research.

Although neurofeminists are committed to challenging and disrupting dominant positivist neuroscientific epistemologies (Bluhm et al., 2012), the use of intersectionality as a guide to reform neuroscientific knowledge production has not been observed until recently. In a publication entitled “Toward a Compassionate Intersectional Neuroscience: Increasing Diversity and Equity in Contemplative Neuroscience,” Weng et al. (2020) propose that the practice of intersectional neuroscience should favor analytical approaches to understanding the brain that “accommodate neural diversity” in accordance with the notion that individual biologies are the product of highly contextualized experiences. To preserve the brain’s individuality but still allow for comparison between subjects, the authors recommend using multi-voxel pattern analysis (MVPA), a multivariate method that uses machine learning to derive brain activity patterns predictive of mental states (Weng et al., 2020). Because this method does not require normalization of brain data and focuses on changes in patterns of brain activity within an individual, the authors argue that MVPA better accommodates the inclusion of “non-normal” brains (Weng et al., 2020). That said, though it avoids normalization of brain activity by focusing on within-subject pattern similarity, MVPA is not “intersectional” *per se* as this approach can be used without any consideration of socio-structural power dynamics or social justice. Weng et al. also contend that intersectional neuroscience should be concerned with conducting research that includes hidden, underrepresented, and marginalized populations and involve a process of “partnering” with participants rather than generating information “about” them. Community-based participatory research reduces power imbalances and generates projects that are rooted in prosocial behavior and empowerment. In combination with the suggestion to use MVPA, a research program co-created with intersecting marginalized populations shifts the focus from the neuroscience of differences to a neuroscience of inclusivity and similarity, both central principles of intersectional research. These approaches to conducting neuroscience facilitate engagement with participants in a way that provides social context to the kinds of generalizations that can be meaningfully drawn from brain data without resorting to harmful reductionism, thereby avoiding or minimizing the kind of distorted “generalization” that arises from ignoring intersectionality. Future work in neurofeminism could benefit from these suggestions for the conduct of intersectional neuroscience.

Neurofeminists have also proposed epistemological frameworks where the relations between knowers and socio-historical contextualization of the phenomenon are constitutive of neuroscientific knowledge. Roy (2018) proposes a multilevel framework of knowledge production, promoting transformative approaches of conducting research that are rooted in feminist theory and activism. Roy envisioned the capacity of researchers to produce socio-historically informed scientific knowledge, even while working within technoscientific and reductionist environments, through a process of knowledge reappropriation and meaning attribution. In her project ‘‘The Co-Production of Knowledge by Reproductive Justice Advocates and Molecular Biologists,’’ Roy used this approach to interrogate women’s reproductive health inequities in light of the NIH policy requiring sex-balanced research^3^. In bringing together neuroendocrinologists and reproductive rights activists, this project highlighted differences in understanding of women’s reproductive health and related policies across knowledge-holders, and demonstrated how creating space for those conversations to take place can generate novel ways for feminists to engage with neuroscience.

Similarly, neurofeminist Gillian Einstein has developed a “situated” approach to neuroscience which parallels the epistemological vision common to this theme of intersectionality research. Einstein (2012) proposed an epistemology which holds that knowledge about the nervous system is “situated” within the multiple hierarchical and socially constructed interactions that involve participants’ experiences, experimenter’s positionality, and technological constraints (Einstein, 2012). This “situated” practice of neuroscience demands that intersecting social identities inform and are informed by varying biologies (Einstein, 2012). Einstein’s “very mixed methods” approach combining qualitative, quantitative behavioral, and quantitative neurophysiological methodologies, was recently used to investigate the multidimensionality of pain experiences in Somali-Canadian women with female genital cutting (Perovic et al., 2021). Importantly, an advisory group from within the participant/target community was created to inform every step of the study development. By combining in-depth interviews about women’s experiences of pain, standardized pain questionnaires, and the physiological assessment of pain in the vulvar region, Perovic et al. (2021) were able to produce novel neuroscientific knowledge about unique pain experiences that intersected with women’s experiences of immigration and cultural acceptance, and in doing so brought to light important considerations for clinical and health advocacy, thus directly contributing to social justice.

From this brief analysis, we highlight the emergence of novel investigative approaches grounded in intersectionality as way of exploring alternative models of knowledge production that are centered around interdisciplinarity, avoiding undue generalization, minimizing the power imbalance between participant and experimenter, and co-creating research for and with hidden populations. These approaches, in addition to extant feminist epistemic alternatives to scientific knowledge production (e.g. Hammonds and Subramaniam, 2003; Richardson, 2013; Roy, 2018; Jordan-Young and Karkazis, 2019), approaches grounded in participatory designs (e.g. Buchmüller et al., 2011) and epistemic injustice (Fricker, 2007; Donnelly, 2018), challenge the very foundations of the dominant mode of knowledge production in quantitative fields and constitute a rich theoretical and methodological foundation for an intersectional neuroscience of sex/gender.

## Advancing Neurofeminist Research With Intersectionality

Intersectionality is undoubtedly a fertile feminist theoretical framework for many disciplines including neuroscience, particularly as scientific narratives around women’s brains and the brains of sex/gender-non-conforming people tend to be essentialized and decontextualized (Fine, 2010; Bluhm et al., 2012; Dussauge, 2014; Joel and Vikhanski, 2019; Jordan-Young and Karkazis, 2019; Rippon, 2019; Llaveria Caselles, 2021). In focusing on three themes of psychological research informed by intersectionality, this analysis identifies specific areas, practices, and critical positions that have the potential to advance the feminist practice of neuroscience.

With regard to theme one, which described intersectionality-informed research on health inequality, we identify the following main areas for advancement: First, neurofeminism will benefit from shifting focus to engage in neuroscientific research that is *systems-centered*, wherein oppressive social structures impacting inequalities in sex/gender-related brain health are modeled and tested. The operationalization and integration of social-structural variables in understanding sex/gender differences in brain health leaves less room for reductive, essentialist explanations that risk inadvertently reinforcing oppressive structures. This approach may also facilitate the connection between our understanding of brain health equality and the need for social change. Incorporating policies and practices, occupations, laws, familial organization, migration status, racial minority status, economic characteristics, etc. into neuroscientific research designs not merely as demographic variables of description but as intersected categories of study will make it possible to empirically demonstrate impacts of social inequality within neuroscience. Police arrests, incarceration history, access to social security, and neighborhood characteristics are a few examples of variables that could be included in order to model and test effects of social structures on health or other outcomes. A second area of advancement is to begin adopting research designs that explicitly contrast privileged and targeted groups assessed before and after the implementation of certain policies, services, or appearance/disappearance of organizations (for more insights on research designs centered on social structure see Krieger, 2019), as high-quality longitudinal analysis can be a big step forward in understanding the impact of socio-structural factors on health inequality. Finally, as the availability of a large brain datasets with greater socio-structural resolution increases, socio-structural causal models will become feasible – although, of course, big data analysis should not be regarded as the final approach to capture intersectionality and diversity since sex/gender and race biases harbor their own risks (Fitsch et al., 2021). Focus should be placed on hidden/invisible populations, and on elucidating how intersecting social group memberships can push individuals into vulnerable positions (del Río-González et al., 2021). Some of this work has already been initiated by neurofeminist scholars (e.g., Somalian immigrant women in Canada with FGC; Perovic et al., 2021), but this work needs to be further expanded.

Against the backdrop of theme two, which discussed research that aims to understand the psychological processing of intersecting group memberships, we identified the following main areas for advancement: First, neurofeminist researchers should place focus on understanding the nuanced interdependence of intersecting identity categories and how these categories can or should be operationally defined. Neurofeminism’s current consideration of sex/gender is explicit, rationalized, and extensively grounded both conceptually and empirically. Conversely, the neurofeminist analysis of sex/gender as interdependent with other social categories such as race is at present often submerged – and concerning face recognition research even being taboo (Kuria, 2014; Kaiser Trujillo et al., forthcoming). In order to avoid treating social categories as homogenous and fixed, neurofeminist research must be dedicated to interrogating and challenging the operationalization of such categories (Marecek, 2016). Second, consideration for the socio-structural interdependence of social categories must be contextualized within systems of privilege and oppression. For instance, developing studies that manipulate power dynamics related to group memberships could provide new insight into the brain processing of sex/gender. As well, adopting an approach like that of Chaney et al. (2020), where the processing of social group membership is considered together with *who* is processing these social cues, can open new avenues for a socio-historically situated sex/gender neuroscience.

Finally, the third theme of research elucidates a more fundamental potential division between intersectional and conventional neuroscience perspectives, particularly in regard to the roles of experimenter and participant, and their involvement (or lack thereof) in the production of knowledge. Considering how to reconcile epistemological disagreements between these two frameworks highlights a clear and pressing need for an expansion of interdisciplinary approaches to neuroscience research that employ mixed methods, consider principles of inclusivity and diversity in morphometrical neuroscientific measures over “normalization,” and demand reflection on the socio-historical situatedness of not only the participants but also the researchers and the research itself. To date, epistemological propositions made by neurofeminists such as Roy (2018) and Einstein (2012) align well with an intersectional perspective and can also generate novel neurofeminist investigative avenues, but more research using these perspectives remains to be done. Additionally, as the neurofeminist field grows it will be crucial to expand means of enhancing awareness around the importance of recognition and inclusion of this type of research and the scholars conducting it in mainstream literatures. Initiatives such as the Neurogenderings Network^4^ have been developed in response to epistemological exclusionary practices, and can be instrumental in preventing epistemic oppression and erasure. Together, these avenues of promoting an awareness as to the situated nature of scientific knowledge and the plurality of knowledge holders open up numerous avenues of future direction for the field of neuroscience.

## Limitations

While our analysis allowed us to identify where intersectionality can advance neurofeminist research, it is also subject to several limitations. First, our categorization of intersectionality research within the three themes identified was conducted to facilitate the present analysis and should therefore itself be considered as contextually situated within a discussion of neurofeminism rather than as an absolute or exhaustive taxonomy of intersectionality research. Second, situating our analysis using psychology as a background framework, we certainly narrow the interdisciplinary focus that neurofeminism champions. Neurofeminism is informed by several disciplines, some of which themselves already conduct epistemic, ethical, and critical race analyses. Thus, future research must work to further unearth the specific epistemic differences and overlap between interdisciplinary approaches to knowledge production in psychology and elsewhere. For instance, the body of clinical and biomedical research grounded in intersectionality is growing, and may certainly provide insights for neuroscience (Hankivsky et al., 2017). Clearly, intersectionality’s explicit focus on social change will be of benefit for neurofeminism through widening the sex/gender-centered scope of this community. We are aware that for some scholars, aspects of the research themes we highlight here may not be considered truly intersectional research. Similarly, when discussing neurofeminism and its position with respect to intersectionality, we purposely aim to reflect more broadly on the research, but recognize that in this approach may have overlooked some relevant neurofeminist and intersectional research.

## Conclusion

Intersectionality can contribute to advancing neurofeminist research and practices in the study of sex/gender. Due to its capacity to expand our understanding of sex/gender into a broader landscape of social categories, incorporating approaches from intersectionality can inform the study of these categories while promoting research that measures or otherwise accounts for their interdependency rather than falsely orthogonalizing them. Further, intersectionality’s focus on social justice, discrimination, and equality resonates with the core fundaments of neurofeminism. However, neurofeminism, a field operating within the neurosciences, is closely bound to the scientific method, and as such any neurofeminist research incorporating intersectionality must critically consider its own methodological and socio-historical situatedness in order to minimize the risks of biologizing and essentializing intersected identity categories and thereby undercutting the social-justice-oriented goals of the endeavor.

## Author Contributions

Both authors listed have made a substantial, direct and intellectual contribution to the work, and approved it for publication.

## Conflict of Interest

The authors declare that the research was conducted in the absence of any commercial or financial relationships that could be construed as a potential conflict of interest.

## Publisher’s Note

All claims expressed in this article are solely those of the authors and do not necessarily represent those of their affiliated organizations, or those of the publisher, the editors and the reviewers. Any product that may be evaluated in this article, or claim that may be made by its manufacturer, is not guaranteed or endorsed by the publisher.

## Footnotes

1 For decades, empirical and theoretical evidence has shown that biological “sex” cannot be empirically disentangled from “gender” due to the complex embeddedness of these constructs (e.g., Haraway, 1992; Butler, 1993; Fausto-Sterling, 2000). Not only are these two constructs entangled, the generic use of each for describing a “biological” versus a “social” phenomenon instead of using the actual biological measure of the social construct, is unscientific. In other words, using “sex” instead of, for instance, “estradiol” or using “gender” instead of, for instance, “gendered attitude” has become far too unprecise based on the knowledge that gender studies and biology have provided. Thus, using “sex” and “gender” separately perpetuates, terminologically and epistemologically, a duality which, first, is not a clear-cut binary and, second, is conceptually erroneous given the diversity captured within and across these terms. In the present analysis we employ the hybrid term sex/gender (Fausto-Sterling, 2012; Kaiser, 2012; Schellenberg and Kaiser, 2018) to describe this embeddedness and overcome the apparent dichotomy. Importantly, the use of sex/gender as a hybrid term is not to be interpreted as an argument that “sex” and “gender” are reducible to the same thing. To suggest that “sex” and “gender” are reducible to the same thing would certainly be counter to any notion of an “intersection” between the two.

2 A theoretical framework is defined as a theory that can support the development of other theories, methods, and research questions (Imenda, 2014). Similar to Else-Quest and Hyde (2016a), we refer to intersectionality as a series of assumptions/principles and commitments comprising a theoretical *framework* that can be employed to inform research and knowledge production more broadly, rather than as a falsifiable theory (Else-Quest and Hyde, 2016a).

3
http://wgss.emory.edu/RoyLab/

4
https://neurogenderings.wordpress.com/
